# Supplementary material for: A simple method for the calculation of dialysis Kt factor as a quantitative measure of removal efficiency of uremic retention solutes: Applicability to high-dialysate vs low-dialysate volume technologies
Source: PLoS One. 2020 May 29;15(5):e0233331. doi: 10.1371/journal.pone.0233331 (PMC7259768; doi:10.1371/journal.pone.0233331)
Supplement: S1 File — Calculation of urea distribution from quantity in spent dialysate and changes in plasma levels. (DOCX) [file pone.0233331.s001.docx]

**Appendix: Calculation of urea distribution volume from quantity in spent dialysate and changes in plasma levels.**

Let’s assume:

Q= urea quantity in spent dialysate

V_app_= apparent urea distribution volume without correction of Q for ultrafiltration component

V_1_= Urea distribution volume at dialysis start (assumedly total water volume at dialysis start)

V_2_= Urea distribution volume at dialysis end (assumedly total body water at dialysis end, i.e. “V”)

UF= convective fluid losses along dialysis, driving urea removal without changes in concentration (neglecting osmotic water fluxes, UF=V_1_-V_2_): may be positive, zero or negative (i.e. net back-filtration)

P_1_= urea pw concentration at dialysis start

P_2_= urea pw concentration at dialysis end (after equilibration, see Methods); it is supposed that P_2_<P_1_ as an effect of dialysis; urea generation is disregarded in the relatively short dialysis time

ΔP= (P_1_-P_2_)

then:

V_app_=Q/(P_1_-P_2_) (1)

Q=P_1_*V_1_-P_2_*V_2_=P_1_*V_1_-P_2_(V_1_-UF)=(P_1_-P_2_)*V_1_+P_2_*UF (2)

Equation 1 then becomes:

V_app_=V_1_+P_2_*UF/(P_1_-P_2_) (3)

Equation 2 can also be solved as:

Q=P_1_(V_2_+UF)-P_2_*V_2_=(P_1_-P_2_)*V_2_+P_1_*UF

and equation 1 then becomes

V_app_=V_2_+P_1_*UF/(P_1_-P_2_) (4)

Solving for V_2_=V:

V=V_app_ – P_1_*UF/(P_1_-P_2_) = Q/ΔP - P_1_*UF/ΔP (5)

One can observe that if UF is zero, UF/ΔP is also zero and V=V_app_=V_1_

If UF is positive, V<V_1_, P_1_*UF/ΔP is positive, and V<V_app_; since also P_2_*UF/ΔP is positive, then V_1_<V_app_, so that V<V_1_<V_app_

If UF is negative, V>V_1_, P_1_*UF/ΔP becomes negative and V>V_app_; since also P_2_*UF/ΔP is negative, then V_2_>V_app_ so that V>V_1_>V_app_
