# Supplementary material for: A simple method for the calculation of dialysis Kt factor as a quantitative measure of removal efficiency of uremic retention solutes: Applicability to high-dialysate vs low-dialysate volume technologies
Source: PLoS One. 2020 May 29;15(5):e0233331. doi: 10.1371/journal.pone.0233331 (PMC7259768; doi:10.1371/journal.pone.0233331)
Supplement: S2 File — (DOCX) [file pone.0233331.s002.docx]

**S2 File: Correlation analysis full results**

Figure 2: Kt_inst_ (y) vs Kt_bal_ (x)

- Urea: y = 1,0946x + 1,8358, R² = 0,922, r=0.96
- Creatinine: y = 1,1675x + 0,6202, R² = 0,8186, r=0.90
- Phosphorus: y = 1,2898x - 0,2453, R² = 0,9404, r=0.97
- b_2_M: y = 1,7156x - 2,0986, R² = 0,9421, r=0.97

Figure 3: Q_inst_ (y) vs Q_bal_ (x)

- Urea: y = y = 1,0364x - 0,422, R² = 0,7226, r=0.85
- Creatinine: y = 0,9641x + 76,952, R² = 0,8811, r=0.94
- Phosphorus: y = y = 0,9846x + 56,497, R² = 0,8781, r=0.94
- b_2_M: y = 0,8625x + 14,62, R² = 0,9429, r=0.97

S1 Figure: Kt_cor_ (y) vs Kt_bal_ (x)

- Urea: y = 0,8529x + 5,0503, R² = 0,7546, r=0.88
- Creatinine: y = 0,8366x + 3,715, R² = 0,8663, r=0.93
- Phopsphorus: y = 0,8018x + 4,474, R² = 0,8617, r=0.94
- b_2_M: y = 0,8767x + 0,5619, R² = 0,9357, r=0.97

S2 Figure: - eKt/V_Daug_ (y) vs eKt/V_bal_ (x), y = 0,9913x - 0,0317, R² = 0,983, r=0.99

- KtDaug (y) vs Ktbal(x): y = 1,0429x - 2,2599, R² = 0,989, r=0.99
