# Supplementary figures and images for: A simple method for the calculation of dialysis Kt factor as a quantitative measure of removal efficiency of uremic retention solutes: Applicability to high-dialysate vs low-dialysate volume technologies
Source: PLoS One. 2020 May 29;15(5):e0233331. doi: 10.1371/journal.pone.0233331 (PMC7259768; doi:10.1371/journal.pone.0233331)

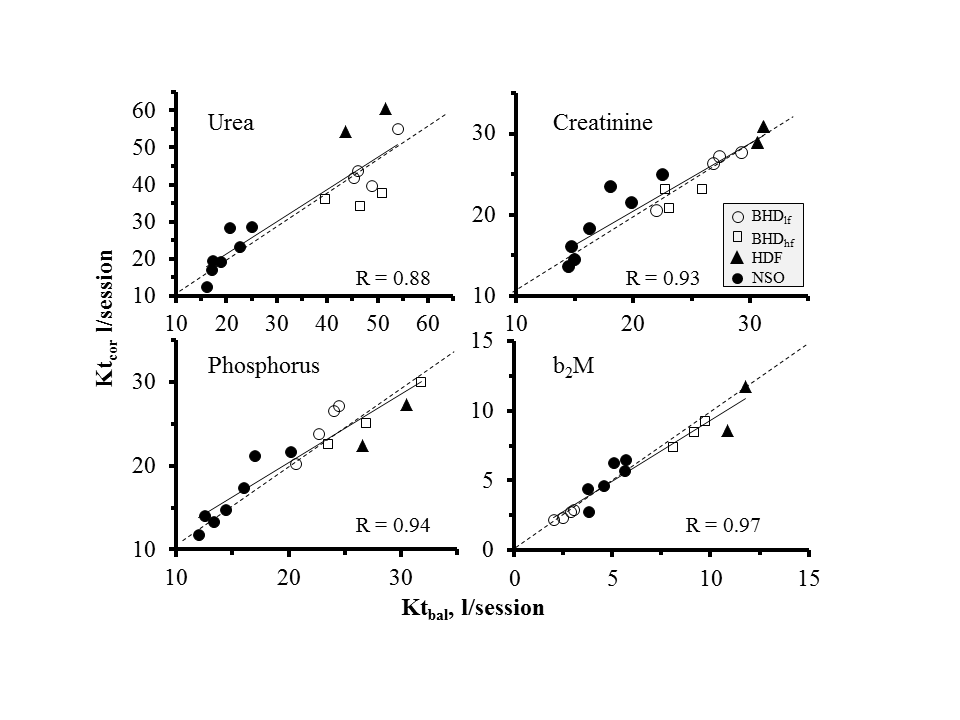

Supplement: S1 Fig — Ktcor is calculated from Qinst and TACbal, as explained in the text. Individual data is shown for urea, creatinine, phosphorus and b2M. Correlation equation lines (full) and coefficients, and identity lines (interrupted) are indicated. Treatment modality is indicated in the inset. (TIF) [file pone.0233331.s003.tif]

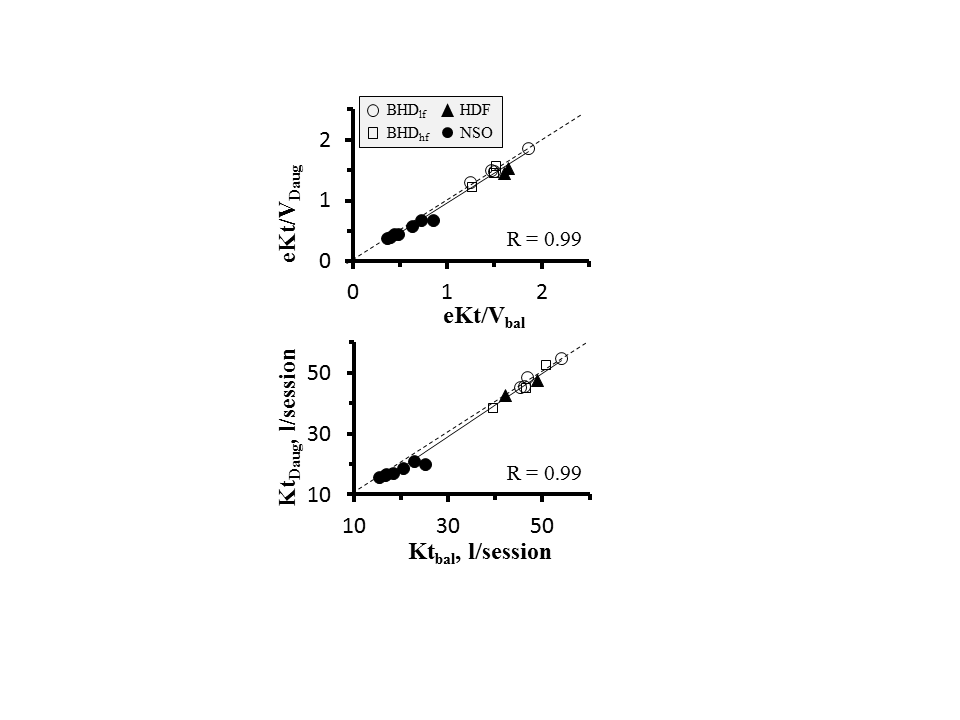

Supplement: S2 Fig — eKt/VDaug and KtDaug indicate values derived by Daugirdas’ second generation equation, eKt/Vbal and Ktbal refer to our calculations in full balance studies. Correlation equation lines (full) and coefficients, and identity lines (interrupted) are shown. Treatment modality is indicated in the inset. (TIF) [file pone.0233331.s004.tif]
