# Supplementary material for: A simple method for the calculation of dialysis Kt factor as a quantitative measure of removal efficiency of uremic retention solutes: Applicability to high-dialysate vs low-dialysate volume technologies
Source: PLoS One. 2020 May 29;15(5):e0233331. doi: 10.1371/journal.pone.0233331 (PMC7259768; doi:10.1371/journal.pone.0233331)
Supplement: S1 Table — In parenthesis is the ratio of mean Kt in different modalities against corresponding BHDlf mean, taken as reference. Ratio to urea expresses the ratio of individual non-urea solute Kt against Kt of urea within each dialytic modality for each study (mean±SD). Statistical level of differences is indicated below the table. (DOCX) [file pone.0233331.s005.docx]

Table S1. Weekly Kt (mean±SD, liters) of urea, creatinine, phosphorus and β_2_M with different dialytic modalities and calculations (“bal” and “inst”). In parenthesis is the ratio of mean Kt in different modalities against corresponding BHD_lf_ mean, taken as reference. Ratio to urea expresses the ratio of individual non-urea solute Kt against Kt of urea within each dialytic modality for each study (mean±SD)

|  | BHD_lf_ |  | BHD_hf_ |  | HDF |  | NSO |  |
| --- | --- | --- | --- | --- | --- | --- | --- | --- |
|  | bal | inst | bal | inst | bal | inst | bal | inst |
|  |  |  |  |  |  |  |  |  |
| Urea | 146.1±14.0 | 151.1±14.0 | 137.1±17.0 (0.94) | 160.2±5.2 (1.06) | 136.7±14.4 (0.94) | 174.6±1.0^&^ (1.15) | 116.2±21.6° (0.79) | 133.9±23.0 (0.89) |
|  |  |  |  |  |  |  |  |  |
| Creatinine | 79.1±9.2 | 83.8±11.8 | 71.8±5.1 (0.91) | 94.7±7.0 (1.13) | 92.7±1.2^#^(1.17) | 119.4±11.9°(1.42) | 103.7±18.1°°(1,31) | 123.7±18.8°° (1.48) |
| -ratio to urea | 0.55±0.07 | 0.56±0.06 | 0.53±0.07 | 0.59±0.02 | 0.69±0.08 | 0.66±0.10 | 0.89±0.02* | 0.93±0.08* |
|  |  |  |  |  |  |  |  |  |
| Phosphorus | 68.8±5.1 | 86.0±9.6 | 82.4±12.5(1.20) | 100.4±12.9 (1.17) | 85.6±8.1° (1.24) | 121.9±7.5° (1.42) | 90.4±17.4° (1.31) | 114.6±16.5° (1.33) |
| -ratio to urea | 0.48±0.4 | 0.57±0.04 | 0.60±0.08° | 0.63±0.07 | 0.63±0.01^$^ | 0.67±0.07° | 0.78±0.04* | 0.86±0.05* |
|  |  |  |  |  |  |  |  |  |
| b_2_M | 7.7±1.4 | 7.6±0.7 | 27.1±2.5^$^ (3.52) | 35.7±4.3^$^ (4.69) | 33.9±1.9^$#^ (4.40) | 56.9±10.2^$#^(7.46) | 28.4±5.2^$^ (3.69) | 36.5±9.4^$^ (4.80) |
| -ratio to urea | 0.05±0.01 | 0.05±0.01 | 0.20±0.03^$^ | 0.22±0.02^$^ | 0.25±0.01^$^ | 0.31±0.04^$#^ | 0.24±0.02^$^ | 0.26±0.05^$^ |

“Balance” data:

° p<0.04 vs BHD_lf_; ^#^p<0.02 vs BHD_hf_; °°p<0.04 vs BHD_lf&hf_; *p<0.01 vs BHD_lf&hf_ and HDF; ^$^p<0.01 vs BHD_lf_;

“Instant” data:

& p<0.04 vs BHD_lf&hf_ and NSO; °°p<0.04 vs BHD_lf&hf_; °p<0.02 vs BHD_lf_; *p<0.01 vs BHD_lf&hf_ and HDF; °°°p<0.01 vs BHD_lf&hf_; #p<0.05 vs BHD_hf_; ^$^p<0.01 vs BHD_lf_;
