# Supplementary material for: A simple method for the calculation of dialysis Kt factor as a quantitative measure of removal efficiency of uremic retention solutes: Applicability to high-dialysate vs low-dialysate volume technologies
Source: PLoS One. 2020 May 29;15(5):e0233331. doi: 10.1371/journal.pone.0233331 (PMC7259768; doi:10.1371/journal.pone.0233331)
Supplement: Attachment — Submitted filename: Response to Reviewers.docx [file pone.0233331.s007.docx]

PONE-D-20-05583

A simple method to measure the Kt factor for comparing removal efficiency of uremic retention

solutes with different dialytic technologies (Changed to: A simple method for calculation of dialysis Kt factor as a quantitative measure of removal efficiency of uremic retention solutes: applicability to high-dialysate vs low-dialysate volume technologies)

PLOS ONE

**Response to Reviewers**

We thank the Reviewers for appreciation of our work and time spent in giving comments and suggestions; we are here detailing actions taken for each individual point. For a better differentiation, responses are in blue. Cross-references to text lines refer to “manuscript with track changes”.

**Reviewer #1**:

1. In line 55-58, calculation of the Kt factor is described. However, when you divide the total

quantity removed by time-averaged plasma concentration (TAC) you obtain a plasma clearance

in mL/min if I’m correct, whereas previously it was stated that the Kt factor is the plasma

volume totally cleared of any solute. Please clarify.

Kt is dimensionally “volume” (the plasma volume cleared in a specified time):

Kt = clearance*time = volume/time*time = volume; this is the same as:

quantity/TAC = quantity/(quantity/volume) = volume.

Conceptually it may be taken as a clearance value where the usual time unit (min) is integrated over a given time (i.e. duration of dialysis treatment). We have not made any changes to text, since we think that calculations in Methods allow to check for dimension of each term.

2. Instant D/P and Kt were significantly higher compared with the balanced D/P and Kt. As

balanced values are more accurate, and instant and balanced measurement were correlated,

would it be possible to apply a mathematical correction to approximate balanced metrics more

accurately using only instant measurements? This would reduce blood and dialysate sampling

frequency, improving the clinical applicability of this method.

We were tempted indeed to do as suggested, but we didn’t for several reasons: the ratio of Kt_inst_ to Kt_bal_ differs in relation to methodology and solutes (it is intuitive that such differences derive from the efficiency of individual solute removal, which varies with membrane permeability and solute characteristics). As an example, “inst” and “bal” Kt of b_2_M were equal in low-flux BHD, where no systemic disequilibrium occurs, but not in high-efficiency treatments (see Fig3). So introduction of “correction factors” for “bal” calculations from “inst” data would result in more complexity and possible biases. We think that a more simple and reliable method for extrapolation of “bal” data from “inst” ones is that suggested in the paper, i.e. calculation of “quantity” from “inst” and “TACbal” with 2 additional blood samples. Please note that both calculations are “accurate”, they simply have a different meaning.

According to these reasoning, we have been reluctant to follow Reviewer’s suggestion, and have made no changes to data presentation in the article. We hope the Reviewer will agree too.

3. To facilitate the interpretation of figures, I would recommend to include a legend on the right side of the figure explaining the meaning of bars and symbols, replacing or complementing the explanation in the legend.

We have made appropriate changes in figures and legends according to this suggestion.

4. If Kt metrics would be used in the clinics to assess dialysis adequacy, targets must be

available to guide clinicians on how to adjust the dialysis prescription. Therefore,

standardization of Kt for distribution volume/bodyweight/BSA is required. Therefore, the

method for calculation of Kt presented in this study is not usable for evaluating dialysis

adequacy in patients yet. Although the authors state that Kt can be used for comparison of

dialysis technologies/modalities, this should be pointed out more clearly in the discussion.

The main aim of our paper is to show the feasibility of a simple methodology for direct calculation of Kt of urea or other solutes, a rather novel measure of dialysis dose; its applicability as an “adequacy” index and need of “normalization” remain to be explored. Extensive investigations by Lowrie and coll in past decades (see References 12-15 among others) indicate that “unnormalized” single session urea Kt is per sè a strong predictor of mortality; they have also suggested that correction for “V” factor may expose small-size people to under-dialysis. For this reason we have elected to present our data as “absolute” measures, implying that any normalization may be independently undertaken according to need.

We have indeed shown that Kt calculation does detect significant differences, both qualitative and quantitative, in removal efficiency between methodologies (all assumed to represent “standard of therapy”), and that the simplified calculation is reliable. Thus, it is true that Kt metrics is a new instrument for comparisons between methodologies. This is our practical message. We have better expressed this concept in Discussion with the following statements: “Target single session urea Kt values have already been suggested in tabular form as possible indices of dialysis adequacy [14, 15], but for other solutes those limits have to be defined. Irrespective of any adequacy meaning, Kt metrics represents a novel, powerful instrument for quantitative comparisons between methodologies, and may be applied to check at a patient level, rather than in “ex-vivo” simulations, performance of the many technologies and dialysis methodologies already in clinical use or forthcoming”. (Discussion, lines 390-396).

5. Discussion: it is repeatedly stated that Kt may be measured in the clinical settings for “any”

solute removed by dialysis. However, only small and middle molecular weight uremic toxins

were evaluated in the present study, and protein-bound uremic toxins (PBUTs) were not. In

contrast to small and middle molecular weight uremic toxins, D/P (using the free PBUT fraction

for plasma concentration) may not remain stable for PBUTs as the equilibrium between the free

and bound PBUT fraction shifts towards the bound-fraction during a dialysis session. If possible,

you could consider measuring PBUTs if storage samples are available. Otherwise, please limit

the statement to small and middle molecular weight toxins.

This is a complex and well taken point. Theoretically, since PBUTs are small molecules (m.w. 100-200), D/P ratio (relative to the free fraction) is expected not to change along the run despite predicted changes in binding dynamics. The main concern we envisage is detectability of these compounds in dialysate (free fraction concentration in blood is not far from the detection limit). Since we have no data on this point, we have added to “limitations” of the study these statements: “Additionally, dialysate-side calculations (both “inst” and “bal”) are expected to underestimate total removal of peptides/small proteins being absorbed to dialyzer membranes, however small this phenomenon may be [4]. Lastly, we have not checked for D/P stability along the run and detectability in dialysate of poorly diffusible, tightly protein-bound solutes, blamed of significant uremic toxicity. Due to their putative relevance, further investigation on extendibility of our methodology to these compounds appears worthwhile”(lines 379-385). The next statement now reads: “The strength of our data is that it allows in the real world practice a simple and reliable quantitative measure of the efficiency with which any free plasma solute, as long as it is detectable in the dialysate, is removed by dialysis itself without the need of whole dialysate collection” (Lines 385-388). As for PBUT dosage, it is actually unavailable in our Hospital.

6. English editing (language and grammar) is required

Thank you for the advice. Our English style has so far satisfied the requirements of not few scientific journals, but we are open to any indications from the Editorial Office.

**Reviewer #2:**

(1) The paper compares NSO treatments (with high flux filters) versus BHD/HDF

treatments (both with low flux and high flux, with and without ultrafiltration). The title of the

paper could also have been "differences between NSO and in-center HD modalities". Apparently the authors selected the frequency of the treatment as main difference, but it can be argued that the main differences in treatment are the use of low flux versus high flux filters, or the

amount of ultrafiltration. Next to (or instead) a comparison between NSO and BHD/HDF a comparison between low flux and high flux filters could (should) have been presented and discussed in more detail (for all the measured toxins urea/creat/phosphate/b2m).

The main difference between methodologies, rather than flux, was the operational context, low dialysate/high saturation (NSO) and high dialysate/low saturation (BHD and HDF), with expected (and verified) great differences in D/P ratio. In addition the main aim of our paper, rather than any comparison, was to show the feasibility of measurement of Kt, as a novel dialysis metrics, in a simplified way, avoiding full balance performance. So, we get the Reviewer’s suggestion to change the paper title in a slightly different way: “A simple method for calculation of dialysis Kt factor as a quantitative measure of removal efficiency of uremic retention solutes: applicability to high-dialysate vs low-dialysate volume technologies”

Please note that low flux/high flux has no role in D/P for common low m.w. solutes, differences occurs only for high-m.w. solutes (e.g. b_2_M), as we have already published (see Ref 4). Even ultrafiltration does not consistently affect low-m.v. solute D/P. These data are clearly apparent in Fig1. As for comparison of Kt in different methodologies, our main target was rather to show reliability of simplified (i.e. “inst”) calculations vs full balances. Though, comparisons between methodologies and membranes have been done and shown in Table S1, already provided, and discussed in relation to the non-proportionality of removal of individual solutes, as shown in Fig4.

We hope the Reviewer will find his points adequately dealt with.

(2) It is not entirely clear how the "inst" values (Ktinst, Qinst) and "bal" values (Ktbal, Qbal)

have been derived. A better explanation than the given "intra-session mean of instant points"

and "calculated values form full balance data" well help the reader to understand what these

values really mean.

We understand that the Reviewer refers to the legend in Fig1. We have modified it with the following: “D/P ratios of urea, creatinine, phosphorus and b_2_M are shown as instant data at different treatment times, as mean of all intra-session values (Mean), and as corresponding values in full balance studies (Bal)”(lines 175-177). In the Methods section definitions were given: “Kt (liters/session) in full balances (Kt_bal_) was calculated as the ratio of measured quantity of each solute X in spent dialysate (Q_bal_) and corresponding TAC_bal_ . In instant calculations, Kt (Kt_inst_) was instead the product of measured instant D/P (either as individual value at 60 min in NSO or the mean of the 3-4 serial determinations in BHD/HDF) by dialysate volume”(lines 127-130)

(3) It is recognized that "Kt" is better measure to compare the effectiveness of a specific

dialysis treatment (such as BHD, HDF, NSO, PD) instead of "Kt/V". But as a measure of dialysis

dose to a specific patient "Kt/V" has to be preferred. Perhaps it is better to talk about Kt as

"absolute dialysis dose" and Kt/V as "normalized dialysis dose".

This was indeed our supposition, and we are sorry that this concept didn’t clearly emerge from the article. We have added these concepts in the discussion (lines 309 and 311). For Kt as a dialysis dose, please refer to answer to point 4 of the Reviewer #1.

4) The discussion paragraph is quite long and extensive. For clarity and readiness it would be

better to condense and to focus on the main points.

We have shortened the Discussion as much as possible, several non-essential sentences have been deleted.

We hope that our answers and actions will fully satisfy Reviewers’ expectations.

As for the “Journal requirements” indications, please refer to the cover letter
